# Characterization and Pharmacological Properties of a Novel Multifunctional Kunitz Inhibitor from *Erythrina velutina* Seeds

**DOI:** 10.1371/journal.pone.0063571

**Published:** 2013-05-28

**Authors:** Richele J. A. Machado, Norberto K. V. Monteiro, Ludovico Migliolo, Osmar N. Silva, Michele F. S. Pinto, Adeliana S. Oliveira, Octávio L. Franco, Sumika Kiyota, Marcelo P. Bemquerer, Adriana F. Uchoa, Ana H. A. Morais, Elizeu A. Santos

**Affiliations:** 1 Laboratório de Química e Função de Proteínas Bioativas, Departamento de Bioquímica, Centro de Biociências, Universidade Federal do Rio Grande do Norte, Natal, RN, Brasil; 2 Departamento de Nutrição, Centro de Ciências da Saúde, Universidade Federal do Rio Grande do Norte, Natal, RN, Brasil; 3 Centro de Análises Proteômicas e Bioquímicas, Programa de Pós-Graduação em Ciências Gênomicas e Biotecnologia, Universidade Católica de Brasília, Brasília, Brasil; 4 Laboratório de Espectrometria de Massa, Embrapa Recursos Genéticos e Biotecnologia, Brasília, Brasil; 5 Laboratório de Bioquímica de Proteínas e Peptídeos, Centro de Pesquisa e Desenvolvimento de Sanidade Animal, Instituto Biológico, São Paulo, Brasil; 6 Departamento de Biologia Celular e Genética, Centro de Biociências, Universidade Federal do Rio Grande do Norte, Natal, RN, Brasil; NIAID, United States of America

## Abstract

Inhibitors of peptidases isolated from leguminous seeds have been studied for their pharmacological properties. The present study focused on purification, biochemical characterization and anti-inflammatory and anticoagulant evaluation of a novel Kunitz trypsin inhibitor from *Erythrina velutina* seeds (EvTI). Trypsin inhibitors were purified by ammonium sulfate (30–60%), fractionation followed by Trypsin-Sepharose affinity chromatography and reversed-phase high performance liquid chromatography. The purified inhibitor showed molecular mass of 19,210.48 Da. Furthermore, a second isoform with 19,228.16 Da was also observed. The inhibitor that showed highest trypsin specificity and enhanced recovery yield was named EvTI (P2) and was selected for further analysis. The EvTI peptide fragments, generated by trypsin and pepsin digestion, were further analyzed by MALDI-ToF-ToF mass spectrometry, allowing a partial primary structure elucidation. EvTI exhibited inhibitory activity against trypsin with IC_50_ of 2.2×10^−8^ mol.L^−1^ and constant inhibition (Ki) of 1.0×10^−8^ mol.L^−1^, by a non-competitive mechanism. In addition to inhibit the activity of trypsin, EvTI also inhibited factor Xa and neutrophil elastase, but do not inhibit thrombin, chymotrypsin or peptidase 3. EvTI was investigated for its anti-inflammatory and anti-coagulant properties. Firstly, EvTI showed no cytotoxic effect on human peripheral blood cells. Nevertheless, the inhibitor was able to prolong the clotting time in a dose-dependent manner by using *in vitro* and *in vivo* models. Due to anti-inflammatory and anticoagulant EvTI properties, two sepsis models were here challenged. EvTI inhibited leukocyte migration and specifically acted by inhibiting TNF-α release and stimulating IFN-α and IL-12 synthesis. The data presented clearly contribute to a better understanding of the use of Kunitz inhibitors in sepsis as a bioactive agent capable of interfering in blood coagulation and inflammation.

## Introduction

The coral tree, *Erythrina velutina*, is a widely known species belonging to the *Erythrina* genus, Fabaceae family, Papillionoideae subfamily and known popularly as mulungu. This plant is commonly found in tropical and sub-tropical regions of the World [1]. Currently there are over 130 species of *Erythrina* from which at least 70 are native to the Americas [2]. Some *Erythrina* species are distinguished by their diverse pharmaceutical activities, including *E. mulungu,* which presents anti-inflammatory activity [2]; *E. speciosa,* which shows analgesic, anti-inflammatory and antibacterial activities and *E. variegate*, which clearly demonstrates antiseptic activity [3], [4]. Many studies have attributed some of these activities to proteinaceous proteinase inhibitors [5]–[14]. Such inhibitors naturally occur in living organisms and are able to inhibit peptidases [15], [16]. Many authors have reported various therapeutic activities of these inhibitors [5], [6], [9], [11], [13], [17]. Recently a Kunitz-type chymotrypsin inhibitor from *Erythrina velutina* seeds (EvCI) was purified and showed potential for combating inflammation related disorders (sepsis) and disseminated intravascular coagulation (DIC) [18].

The Kunitz-type inhibitors are proteins constituted by approximately 180 amino acid residues with molecular masses ranging between 18 and 26 kDa, and having a low content of cysteine residues which are involved in formation of one or two disulfide bridges. In general, these inhibitors have only one reactive site. Because of this structural feature, they are known as one-head (or “single headed”) inhibitors [16], [19]. In recent years, several studies have shown that these inhibitors may play important roles in the treatment or for preventing various diseases [20]. Many of these diseases could be derived from massive inflammatory cell activation that leads to immunological problems and excessive activation of the coagulation system, as seen in sepsis syndrome. Sepsis is characterized by a complex interaction between the infectious agent and the host pro-inflammatory and pro-coagulant immune responses [21]. This syndrome could be considered as a major problem for health, as it often leads to treatments in intensive care units, affecting about 750,000 people per year; indeed, more than 210,000 people die from sepsis in the United States per year [21], [22]. There are many studies demonstrating the relationship between inflammation and coagulation [23]–[27]. Moreover, it is observed that severe sepsis, septic shock and endotoxemia could be associated with significant effects on coagulation, fibrinolysis and platelet aggregation, as well as modifications in the hemostasis balance states [25]. In health sciences, enzyme coagulation inhibitors have been used as pharmaceuticals for the treatment of certain diseases related to coagulation cascade disorders [28], [29]. However, the search for new and potent inhibitors is still intense to improve the existing drugs and encompass numerous diseases related to blood clotting and inflammation.

The clinical practice that utilizes peptidase inhibitors in sepsis treatment is restricted to a urinary trypsin inhibitor urinastatin which has been widely used in Japanese patients with inflammatory disorders [30]. Moreover, a neutrophil elastase inhibitor sivelestat showing beneficial effects in sepsis treatment has also been used clinically [31]. Additionally, recombinant human activated protein C (rhAPC) exhibiting anti-inflammatory and anticoagulant activities has shown effectiveness in sepsis treatment [31], [32]. A number of studies have been performed in order to screen novel compounds with anti-inflammatory and anticoagulant properties that could be used in the treatment of sepsis, especially those derived from plants. In this context, this study aims to purify, characterize and investigate a Kunitz-type trypsin inhibitor from *E. velutina* seeds with anti-inflammatory and anticoagulant activities. This inhibitor was also evaluated against microorganisms and in an experimental sepsis model.

## Methods

### Reagents

Bovine pancreatic trypsin, human neutrophil elastase, bovine plasma activated factor X, human neutrophil peptidase 3, bovine pancreatic chymotrypsin, human plasma thrombin, immobilized porcine stomach pepsin, azocasein, N-benzoylarginine-4-nitroanilide (BApNA), N-benzoyl-phenylalanyll-valyl-arginine-4-nitroanilide (Bz-Phe-Val-Arg-pNA), N-methoxysuccinyl-alanyl-alanyl-prolyl-valine-4-nitroanilide (MeOSu-Ala-Ala-Pro-Val-pNA), PBS is 0.15 mol.L^−1^ phosphate buffer with 0.136 mol.L-1 sodium chloride, 0.00268 mol.L^−1^ potassium chloride, 0.00147 mol.L-1 monobasic potassium phosphate and 0.00798 mol.L-1 sodium phosphate dibasic hypohydrated were purchased from Sigma, St Louis, USA. The Kits were PT and APTT were purchased from Wiener lab (São Paulo, SP, Brazil). Hydrochloride xylazine and ketamine were purchased from Koning of Brazil Ltda. α-cyano-4-hydroxycinnamic acid was a Sigma product and was previously recrystallized from aqueous acetonitrile. Immobilized pepsin was from Thermo Scientific (Rockford, USA).

### Animals

Swiss mice *Mus musculus* species of two months old and weighing 30 to 35 g were obtained from the vivarium of the Department of Biochemistry, Universidade Federal do Rio Grande do Norte – UFRN. BALB/c, two months old and weighing 30 g, were obtained from the vivarium of Universidade Estadual de Campinas CEMIB-Unicamp and kept in the animal house of the Universidade Católica de Brasília – UCB. The animals were acclimated with four animals per cage and maintained under photoperiod of 12/12 h, at 25±2°C temperature, with access to food and water *ad libitum.* All experiments involving animals comply with the rules of the ethics committee on animal use (ECAU), Federal University of Rio Grande do Norte, under protocol 009/2010 and by the Research Ethics Committee of the Faculty of Medicine (RECFM) at UnB No. 0177–07. Both ethics committee (ECAU and RECFM) specifically approved this study.

### Purification of the trypsin inhibitor from *Erythrina velutina* seeds

*E. velutina* seeds were peeled and cotyledons were ground at 6°C until a fine grained flour with approximately 40 mesh was obtained. This flour was homogenized at 1∶10 (w/v) in 5.0×10^−2^ mol.L^−1^ Tris-HCl buffer, pH 7.5 under stirring for 3 h, at room temperature. The homogenate was centrifuged at 8000 *g* for 30 min at 4°C. The resulting supernatant was designated as crude extract (CE), which was fractionated by ammonium sulfate precipitation in the range: 0–30%, 30–60%, 60–90%. After each precipitation step, the mixture was kept at 4°C for about 20 h and subsequently was centrifuged at 8000 *g* for 30 min at 4°C. Precipitate was resuspended in 5.0×10^−2^ mol.L^−1^ Tris-HCl buffer, pH 7.5, and submitted to dialysis for approximately 20 h against the same buffer. Around 7 mg of 30–60% fraction named fraction 2 was applied onto a Trypsin-Sepharose affinity column (GE Healthcare, Waukesha, USA) pre-equilibrated with 5.0×10^−2^ mol L^−1^ Tris-HCl buffer, pH 7.5. Non-retained proteins were eluted with the same buffer. Furthermore, retained proteins were eluted with 5.0×10^−3^ mol.L^−1^ HCl and collected in aliquots of 5 mL at a flow rate of 2 mL.min^−1^. Protein elution was monitored at 280 nm. The pooled retained fractions, termed TR (trypsin retained) were dialyzed against distilled water, lyophilized and subjected to trypsin inhibition assays using specific substrate (BApNA). TR was purified by a reversed-phase HPLC using Shimadzu C18 analytical column (0.46 cm×25.0 cm, 5 μm, 300 Å, Shimadzu, Kyoto, Japan), solvent A (aqueous 0.1% TFA), solvent B (60% ACN/0.09% TFA). C18 column adsorbed protein was eluted using a linear gradient of 60 to 70% of solvent B (60% ACN/0.09% TFA) in 30 min, at a flow rate of 1 mL.min^−1^, and peak detection using 220 nm wavelength. Two protein components named as Peak 1 (P1) and Peak 2 (P2) were observed and they were manually collected in order to separate them from the mixture. These components were re-chromatographed separately in the same RP-HPLC experimental conditions using Shimadzu C18 analytical column, solvents A and B, flow rate, peak detection, and a linear gradient of 60 to 70% solvent B for 10 min.

### Quantification of proteins and determination of *Erthryna velutina* trypsin inhibitors molecular masses

Proteins were quantified by the Bradford method [33] using bovine serum albumin (BSA) as a standard. The purity inhibitor degree of different fractions and of the purified inhibitors was determined by SDS-PAGE12.5% as described by Laemmli [34]. An aliquot containing approximately 10 ng protein was applied on the gel. After electrophoresis, proteins were silver stained. Molecular weight markers (Fermantas Life Sciences, Thermo Fischer Scientific, Waltham, USA) were utilized as standard. Furthermore, the purified inhibitors were analyzed by electrospray ionization mass spectrometry (ESI-TOF) by using a microTOF-Q II spectrometer from Bruker Daltonics (Billerica, USA). Aliquots of the EvTI was dissolved in formic acid:ACN:H_2_O (1∶50∶50) (v:v) and then applied to the equipment by direct injection. The spectrum was acquired in the positive mode with capillary voltage of 4.5 kV; detection range of spectra was between 500 and 4000 m/z. For manual calibration of the spectrometer an ESI tuning mix Kit (Agilent technologies, Santa Clara, EUA) was used.

### EvTI stability evaluation

The thermal and pH stabilities of EvTI were determined by the method described by Gomes [35]. EvTI aliquots were incubated for 30 min at 37, 40, 50, 60, 70, 80, 90 and 100°C. After the incubation time, the samples were cooled to 4°C. For evaluation of stability to pH variation inhibitor aliquots were dialyzed against the following buffers: glycine-HCl, pH 2.0 and 3.0; sodium phosphate, pH 6.0 and 8.0, and glycine-NaOH, pH 11 and 12, in which the buffer concentration was 0.1 mol.L^−1^. After 30 min incubation at 37°C samples were dialyzed for about 4 h against Tris-HCl, 5.0×10^−2^ mol.L^−1^, pH 7.5. These treated samples were evaluated for trypsin inhibitory activities by enzymatic assays performed in triplicate [36].

### Antitrypsin activity

Antitrypsin activity was determined by using the specific substrate BApNA [36]. Aliquots of 10 μL solution of bovine trypsin (0.3 mg.mL^−1^ in 5.0×10^−2^ mol.L^−1^ Tris-HCl buffer, pH 7.5) were pre-incubated for 10 min at 37°C with 120 µL of 2.5×10^−3^ mol.L^−1^ HCl solution, 365 μL of 5.0×10^−2^ mol.L^−1^ Tris-HCl buffer, pH 7.5 and a volume of 5 µL containing 2 µg of EvTI was added. After this time the reaction was started by adding 500 µL of 1.25×10^−3^ mol.L^−1^ BApNA. The reaction was continued for 15 min, at 37°C and stopped by adding 120 µL of acetic acid 30% (by volume). The 4-nitroaniline formation was monitored in a spectrophotometer (Ultrospec 2100 pro, GE Healthcare Life Sciences, Piscataway, USA) at 405 nm. Negative controls were performed in the same manner as described above except without the addition of substrate. All assays were performed in triplicate.

### Specificity of EvTI for other serine peptidases

The ability of EvTI to inhibit other serine peptidases including activated factor X bovine plasma, human neutrophil elastase, human plasma thrombin, bovine pancreatic chymotrypsin and human neutrophil 3 peptidase (Sigma, St Louis, USA) was analyzed. Approximately 2 µg of EvTI was used in all inhibition assays. In order to determine the inhibitory activity on 96-well plates for Factor Xa (0.2 U.mL^−1^) a solution of Factor Xa was preincubated with 0.15 mol.L^−1^ PBS buffer, pH 7.4 and EvTI for 10 min at 37°C. After this period, the reaction was initiated by adding a chromogenic substrate for factor Xa (1.0×10^−3^ mol.L^−1^). The reaction was stropped with 30% acetic acid (by volume) after 30 min. The absorbance was measured in a spectrophotometer at 405 nm [37]. The inhibitory activity towards elastase was evaluated by pre-incubation of the EvTI with the enzyme (0.2 μmol.L^−1^) (Sigma-Aldrich, St Louis, USA) and 0.15 mol.L^−1^ PBS, pH 7.4 for 10 min at 37°C. The reaction was started after addition of 5.0×10^−3^ mol.L^−1^ N-methoxysuccinyl-Ala-Ala-Pro-Val-pNA. The reaction was stopped by adding 120 µL of 30% acetic acid (by volume). The formation of 4-nitroanilide was monitored at 405 nm [38]. Inhibitory activity for thrombin was evaluated by pre-incubation of thrombin (a solution at 8.0 NIH.mL^−1^ solubilized in deionized water) in 50×10^−3^ mol.L^−1^ Tris-HCl buffer, pH 7.5 with 0.1 mol.L^−1^ NaCl; EvTI was then added and the reaction solution for 10 min at 37°C. After this period, the reaction was started by adding N-benzoyl-phenylalanyll-valyl-arginine-4-nitroanilide (Bz-Phe-Val-Arg-pNA) (3.0×10^−3^ mol.L^−1^). The reaction was stopped with 120 µL of 30% acetic acid (by volume) after 20 min. The formation of 4-nitroaniline was monitored in a spectrophotometer at 405 nm [39]. The inhibitory activity for the peptidase 3 was assessed by incubating peptidase 3 (0.1 μg.μL^−1^ solubilized in deionized water) in 0.15 mol.L^−1^ PBS buffer, pH 7.4 and EvTI for 10 min at 37°C. After this period, the reaction was started by adding MeOSu-Ala-Ala-Pro-Val-pNA (5 mol.L^−1^). The reaction was stropped with 120 µL of 30% acetic acid (by volume) after 60 min. The formation of 4-nitroaniline was monitored in a spectrophotometer at 405 nm [40]. The inhibitory activity against chymotrypsin was determined using bovine chymotrypsin solution (0.2 mg.mL^−1^) that was pre-incubated with 5.0×10^−2^ mol.L^−1^ Tris-HCl buffer, pH 7.5, containing 2.0×10^−2^ mol.L^−1^ CaCl_2_ and EvTI for 15 min at 37°C. After this time the reaction was initiated by addition of 200 µL of 1% azocasein (w/v). After 30 min, the reaction was stopped by adding 300 µL of 20% TCA (w/v). The reaction mixture was centrifuged at 12,000 *g* for 10 min and the supernatant was alkalinized with 2 mol.L^−1^ NaOH 1∶1 (v:v), and absorbance was measured by spectrophotometer at 440 nm [41]. Assays were performed in triplicate and three independent experiments were conducted.

### Determination of IC_50_ and K_i_ against trypsin

Increasing concentrations of EvTI were incubated with 20 µL (at 1.28×10^−2^ mol.L^−1^ in 5.0×10^−2^ mol.L^−1^ Tris-HCl buffer, pH 7.5) trypsin, and enzyme assay was performed as previously described [36]. Percentage of Trypsin inhibitor for each inhibitor concentration was used to construct a titration curve and determination of the IC_50_. In order to determine the mode of inhibition and inhibition constant (K_i_) data was plotted according to Dixon et al., [42] as being the intercepts between two plot lines using two BApNA concentrations (6.25×10^−4^ mol.L^−1^ and 1.25×10^−3^ mol.L^−1^). The enzymatic reaction velocity (v) was expressed as reaction product optical density (OD) at 405 nm in function of reaction time (h) and volume (mL) (V = DO.h^−1^.mL^−1^). Assays were performed as previously described [36].

### Mass spectrometry analysis and sequencing of EvTI

EvTI molecular mass was determined by using MALDI-ToF MS/MS analysis (UltraFlex III, Bruker Daltonics, Billerica, MA). Purified EvTI was dissolved in a minimum volume of water that was mixed with an α-cyano-4-hydroxycinnamic acid saturated matrix solution (1∶3, v:v), spotted onto a MALDI target plate and dried at room temperature for 5 min. The α-cyano-4-hydroxycinnamic acid matrix solution was prepared at 50×10^−3^ mol.L^−1^ in H2O:ACN:TFA (50∶50∶0.3, v:v:v). Protein average mass was obtained in the reector mode with external calibration, using the Protein Calibration Standard I for mass spectrometry (up to 25.000 Da mass range, Bruker Daltonics, Billerica, MA). In addition, the protein was analyzed by ESI optimization conditions performed by injecting in triplicate a standard solution containing standard calibrates at a concentration of 10 ppm. Protein was analyzed immediately after preparation. The extracted ion chromatogram peak areas obtained for each peptide ion were calculated for EvTI molecular mass. Optimized ESI conditions were ion polarity, positive; nebulizer pressure, 4.4 psi; capillary voltage, 4500 V; gas temperature, 180°C; gas flow, 4 L.min^−1^. After the purity and molecular mass analyze of EvTI, it was reduced and alkylated, and digested with immobilized porcine pepsin in solution. Also, the fraction corresponding to the inhibitor in SDS-PAGE12.5% (see above) with was digested in gel by porcine trypsin. For reduction and alkylation, 50 µg of the purified inhibitor was used. Reduction was performed in 300 µL of 0.1 mol.L^−1^ sodium hydrogen carbonate with 0.05 mol.L^−1^ 1,4-dithiotreitol for 60 min at 70°C, followed by alkylation with 0.1 mol.L^−1^ 2-iodoacetamide in a final volume of 600 µL (0.1 mol.L^−1^ sodium hydrogen carbonate) for 40 min at 37°C. Samples were then filtered (0,22 µm) and the alkylated protein was purified by reversed-phase chromatography Vydac analytical C18 column (250×0,46 cm), HPLC. Experimental conditions were as follows: H_2_O:ACN:TFA (95∶5∶0.1, v:v:v) for 5 min, then a linear gradient to H_2_O:ACN:TFA (5∶95∶0.1, v:v:v) over 65 min; 1 mL.min^−1^ flow rate; 40°C; 250 µL of the solution injected. Pepsin hydrolysis was conducted for 12 h in 100 m mol.L^−1^ sodium acetate buffer, pH 4.5 with immobilized enzyme. The obtained peptides were purified by narrow-bore reversed-phase chromatography. Separation was performed in a 30×2 mm Model XR-ODS column (Shimadzu, Kyoto, Japan) in UFLC system. Experimental conditions were as follows: H_2_O:ACN:TFA (95∶5∶0.1, v:v:v) for 5 min, then a linear gradient to H_2_O:ACN:TFA (5∶95∶0.1, v:v:v) over 30 min; 0.4 mL.min^−1^ flow rate; 40°C; 50 µL of the peptide solution injected. For trypsin digestion, the gel containing the protein band was excised and transferred to microfuge tube. The dye Coomassie Blue was taken from the gel with three washes made with aqueous 30% ethanol (by volume) to bleach completely, followed by other wash with aqueous 50% ACN and 2.5×10^−2^ mol.L^−1^ ammonium carbonate for 15 min. ACN was added and the mixture was allowed to incubate for 10 min under vigorous stirring. After this step the gel was dried at reduced pressure (Centri-Vap, Labconco, Kansas City, USA) for 20 min. To the dried gel pieces it was added a solution of trypsin (33 ng.µL^−1^) in a volume sufficient to cover the gel and these suspensions were maintained in an ice bath for 30 min. A 40 μL volume of a solution of ammonium carbonate (5.0×10^−2^ mol.L^−1^) was then added and the system was incubated for 19 h at 37°C [43]. For MALDI-MS analysis, α-cyano-4-hydroxycinnamic acid (CHCA) at 50×10^−3^ mol.L^−1^ in 0.3% aqueous acetonitrile was employed as matrix. The peptides obtained by EvTI hydrolyses with pepsin and trypsin was mixed with CHCA in a proportion of 1∶3 (v:v) and deposited on to a AnchorChip™ target (Bruker Daltonics, Bilerica, USA) and allowed to crystallize at room temperature. The ionization was performed in the positive reflected mode, with the following instrument voltage parameters: *Ion source 1*: 20.00 kV, *Ion source 2*: 17.65 kV, *Lens*: 7.50 kV, *Reflector*: 22.00 kV, *Reflector 2*: 9.80 kV. Data were recorded in the m/z range from 600 to 4,000. Peptide fragmentation was conducted by the LIF^TM^ methodology [44] with the following instrument voltage parameters: *Ion source 1:* 6.00 kV, *Ion source 2:* 5.25 kV, *Lens*: 3.00 kV, *Reflector 1*: 27.00 kV, *Reflector 2*: 11.80 kV, *LIFT 1*, 19.00 kV, *LIFT 2*: 4.70 kV [44]. The spectra interpretation and peptide sequencing were manually performed by using the FlexAnalysis 3.3 software (Bruker Daltonics, Bilerica, USA).

### *In silico* alignment analyses

The peptide sequences generated were examined and compared to Kunitz-type inhibitor sequences deposited in the NCBI (National Center for Biotechnology Information, www. ncbi.nlm.nih.gov) database using the BLASTp serch tool [45]. EvTI sequences that showed highest identities with other inhibitors were aligned by using the BioEdit computer program v. 7.0.9.0 with the ClustalW multiple alignment tool [46].

### Cytotoxic and hemolytic activities of EvTI

EvTI effects on cell viability were assessed on cells from peripheral human blood. Human heparine blood was obtained from the Hospital of the Catholic University of Brasilia cell collection and stored at 4°C. Collection was obtained with written informed consent. About 1 mL of human blood was incubated with BSA and EvTI at different concentrations (2.6×10^−7^–1.3×10^−6^ mol.L^−1^) for 15 min at 37°C to evaluate possible hematological change by blood cell count (CHCELL 6019 – LABORLAB) [47]. The evaluation of hemolytic activity was performed according to Johansson et al., [48]. Triton X-100, 1% (v/v) was the reference for 100% hemolysis. PBS buffer at 0.15 mol.L^−1^, pH 7.4 was the reference for 0% hemolysis. This study was approved by the Research Ethics Committee of the Faculty of Medicine (RECFM) at UnB No. 0177–07.

### *In vitro* antibacterial activity of EvTI

*Escherichia coli* ATCC8739 and *Staphylococcus aureus* ATCC 25923 were used to evaluate the antibacterial activity of EvTI. This evaluation was performed by testing inhibition of growth by broth microdilution according to NCCLS (National Committee for Clinical Laboratory Standards) [49]. Distilled water was used as negative control, while chloramphenicol at 9.6×10^−6^ to 6.2×10^−4^ mol.L^−1^ range was utilized as antibiotic positive control. The lowest concentration that completely inhibited the bacterial growth was considered the minimum inhibitory concentration (MIC).

### Anticoagulant activity

The anticoagulant activity was determined by of PT (prothrombin time) and APTT tests (Activated Partial Thromboplastin Time) according to the manufacturer's instructions (Wiener lab., São Paulo, Brazil) [50].

### Anti-inflammatory activity and anticoagulant EvTI using the experimental sepsis model and assessment of survival of mice treated with EvTI model of polymicrobial sepsis

The model of sepsis induction was performed according to Ebong [51] using cecal ligation surgery and puncture (CLP). Swiss mice (n = 8) were intraperitoneally anesthetized with 2% xylazine hydrochloride and 10% ketamine hydrochloride. Following a laparotomy with 2 cm midline incision, the cecum was exposed and ligated just below the ileocecal valve. The cecum was carefully isolated and the distal 30% part was ligated. It was then punctured five times with a sterile 22-gauge needle and squeezed to extrude the fecal material from the wounds [51]. Next, it was placed back into the abdominal cavity, which was then closed in layers. All treatments were administered 30 min before sepsis induction. The animals were randomly separated into six groups (treated with NaCl only and no sepsis induction, treated with 0.9% (w:v) NaCl, 1 mg.kg^−1^ heparin, 7.5 mg.kg^−1^ diclofenac, 20 mg.kg^−1^ imipenem, 1 mg.kg^−1^ EvTI, and induction of sepsis). A second sepsis model was also used, by an intraperitoneal inoculation of Gram-positive and Gram-negative bacteria. BALB/c mice (n = 5), intraperitoneally anesthetized with 2% xylazine hydrochloride and 10% ketamine hydrochloride, were intraperitoneally inoculated with 50 µL of bacterial suspension, 25 µL *S. aureus* (5×10^9^ CFU.mL^−1^) and 25 µL of *E. coli* (5×10^7^ CFU.mL^−1^). Treatment with EvTI and imipenem was administered immediately after bacterial inoculation and 6 h after infection. The animals were observed for survival over 24 h. For evaluation of the experimental model of polymicrobial sepsis animals were randomly in three groups divided according to the treatment to be administered (5 animals per group) as the following: 1) infected and treated with 10 mg.kg^−1^ EvTI; 2) infected and treated with 20 mg.kg^−1^ imipenem; 3) infected and treated with distilled H_2_O.

### Evaluation of migration of leukocytes into the peritoneal cavity

In order to evaluate the effects of EvTI on leukocyte migration, about 6 h after sepsis induction, the peritonea of mice were exposed and 5.0 mL of sterile 0.9 (w:v) NaCl solution was injected into the peritoneal cavity. A brief massage was immediately performed for further fluid collection, which was then centrifuged for 10 min at 1500 *g* at room temperature. The supernatant was disposed of and 1 mL of 0.15 mol.L^−1^ PBS, pH 7.4 was added to the precipitate. For evidencing and counting of leukocytes, acetic acid at 4% (by volume) was used and total cells were counted in a Neubauer chamber, under optical microscope. Results were expressed as the number of leukocyte.mm^−3^ [52].

### Differential count of peripheral blood leukocytes

Blood smear technique was used for differential counts, and the slides were stained Quick Kit Panótico (LB, Larboclin®, São Paulo, Brazil) [52]. The cells were examined under an optical microscope, and one hundred cells were counted per slide. Thus the percentage of each cell type in the blood (neutrophils, lymphocytes, eosinophils and monocytes) was obtained.

### Determination of cytoKine levels (TNF-α, IL-6, IFN-γ and IL-12) and nitric oxide

The concentrations of TNF-α, IL-10, IFN-γ and IL-12 in peritoneal fluid were determined by ELISA according to the manufacturer specifications (Peprotech – USA). The production of nitrite in the supernatants obtained from mouse peritoneal washings was evaluated according to Green et al., [53].

### *Ex vivo* anticoagulant activity in plasma of septic mice using the activated partial thromboplastin time (APTT)

Six hours after sepsis induction, mice were anesthetized with 2% xylazine hydrochloride and 10% ketamine hydrochloride for total blood collection. The anticoagulant activity was determined by APTT (Activated Partial Thromboplastin Time) tests according to the manufacturer instructions (Wiener lab., São Paulo, Brazil) [50].

### Statistical analysis

All data represent at least three independent experiments and were expressed as mean ± SD (standard deviation), except where explicitly stated otherwise. Differences between groups were compared by using ANOVA and Tukey Test. Differences were considered significant when p value was less than 0.05. Statistical data were analyzed by GraphPad Prism 5.0 software.

## Results

### Purification of trypsin inhibitor from seeds of *Erythrina velutina*

**Figure 1:**
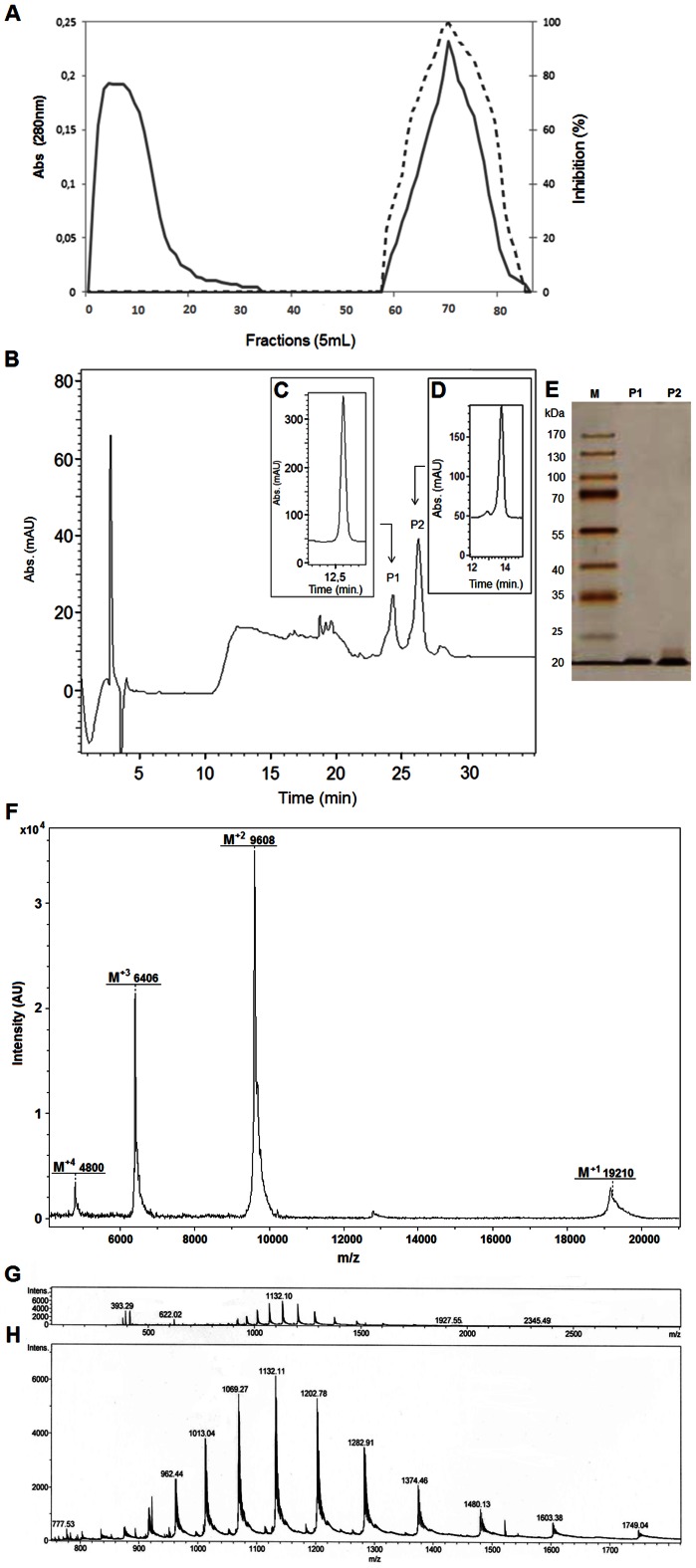
Affinity chromatography on Trypsin-Sepharose of 30–60% ammonium sulfate fraction from *E. velutina* and reversed-phase chromatography of the Trypsin-Sepharose eluates. A. Elution profile of the fraction saturated with 30–60% ammonium sulfate from seed extracts of *E. velutina.* Adsorbed proteins were monitored at 280 nm (―).Inhibitory activity on trypsin (----) was assayed using 50 µL of retained material. B. Elution profile in a reversed-phase column of the retained Trypsin-Sepharose fraction (RT). C and D. Analytical profile of reversed-phase purified trypsin inhibitors. E. Electrophoresis on polyacrylamide gel at 12.5% after developing with silver nitrate. M, Molecular mass markers; EvTI, reversed-phase P1 and P2. F. MALDI Mass spectrometry analysis of EvTI highlighting doble, triple and quadruple charge for molecular mass average of inhibitor. G and H, ESI profile with EvTI overview and zoon analysis showing various inhibitor charges.

Firstly, fraction F2 (obtained by 30–60% ammonium sulfate precipitation) proteins, which showed higher trypsin inhibitory activity, were applied onto a Trypsin-Sepharose affinity column. The retained proteins (TR) were eluted using 5.0×10^−3^ mol.L^−1^ HCl, dialyzed against 5.0×10^−2^ mol.L^−1^ Tris-HCl, pH 7.5, and subjected to the trypsin inhibition assay by using BapNA as substrate (Figure 1A). TR caused complete trypsin inhibition. TR fraction showed two protein bands with apparent molecular masses of 20 and 23 kDa. TR was further purified by HPLC in a Shimadzu analytical column and two protein fractions were observed (P1 and P2) (Figure 1B). The protein fractions obtained were lyophilized and subsequently subjected to trypsin inhibitory assay. Five µg of each peak (P1 and P2) were able to inhibit trypsin at 52%±2.9 and 93%±0.2, respectively. The purified inhibitors were analyzed onto an analytical reversed phase C-18 column. Figure 1 C show P1 with a retention time (RT) of 13 min (when the concentration of ACN in the solvent reached 40.8% B). Figure 1D shows the EvTI (P2) elution profile with retention time of 13.8 C min at 41.3% B. SDS-PAGE analysis revealed that both fractions showed a single protein band with a apparent molecular mass of about 20 kDa (Figure 1E). The inhibitor molecular mass determined by mass spectrometry (UltraFlex III, Bruker Daltonics, Billerica, MA) was 19,210.48 Da (Figure 1F). Moreover, it was possible to identify double (M^2+^), triple (M^3+^) and quadruple (M^4+^) charges for EvTI molecular mass demonstrating the contaminant absence. Moreover ESI (Q-Electrospray microTOF) was further utilized for purity degree analyses demonstrating various ions charged with the presence of M^20+^ (962.44 Da), M^19+^ (1,013.04 Da), M^18+^ (1,069.27 Da), M^17+^ (1,132.11 Da), M^16+^ (1,202.78 Da), M^15+^ (1,282.91 Da), M^14+^ (1,374.46 Da), M^13+^ (1,480.13 Da), M^12+^ (1,603.38 Da) and M^11+^ (1,749.04 Da) charges for EvTI protein (Figure 1H).

### Analysis of the purification steps of the trypsin inhibitors

Table 1 shows the EvTI purification steps with corresponding yield and degree calculated values and results of specific activities. The crude extract (CE) obtained in the first step of the purification process was used as reference for other protein fractions. Table 1 shows that the specific activity of purified EvTI was 1400 IU.mg^−1^, and its purification degree at the final step was 3252 times higher than CE with average recovery of 0.15% of purified EvTI.

**Table 1:** Steps of EvTI purification from *Erythrina velutina seeds*.

Step	Volume (mL)	Total protein (mg)	Total inhibitory activity (UI)	Specific activity (IU.mg^−1^)	Purification (X)	Recovery (%UI)
Crude extract	370	3500	15540	4.42	1	100
F2 (30–60%)	95	1260	16150	12.8	2.8	35
Trypsin-Sepharose	125	11.3	60500	5110	1100	0.30
EvTIb	13	5.3	76180	1400	3200	0.15

IU: inhibitory unit.

### Effect of temperature and pH on the EvTI inhibitory activity

EvTI maintained its inhibitory ability on trypsin catalytic activity in the range of tested pH (2 to 12) and temperature (37 to 100°C) (results shown apart in Figure S1).

### Specificities of EvTI

In order to determine the EvTI specificity of serine peptidases inhibition, enzymatic assays were performed using 2 μg of inhibitor. The results shown in Table 2 demonstrate that EvTI has a higher specific inhibitory activity against trypsin (15.5 IU.mg^−1^) and factor Xa (14.5 IU.mg^−1^) and moderate activity for elastase (1.8 IU.mg^−1^). EvTI showed no significant inhibition for other serine peptidases evaluated (thrombin, chymotrypsin and peptidase 3).

**Table 2:** Results of inhibition specific activities of bovine trypsin, bovine plasma FXa and human neutrophil elastase evaluation by EvTI.

Enzymes	Sources	Specific Activity (UI.mg^−1^)
Trypsin	Bovine pancreas	15.5
FXa	Bovine plasma	14.5
Elastase	Human neutrophil	1.8

IU: inhibitory unit; FXa: Factor X.

### Determination of IC_50_ and K_i_ for trypsin of EvTI

**Figure 2:**
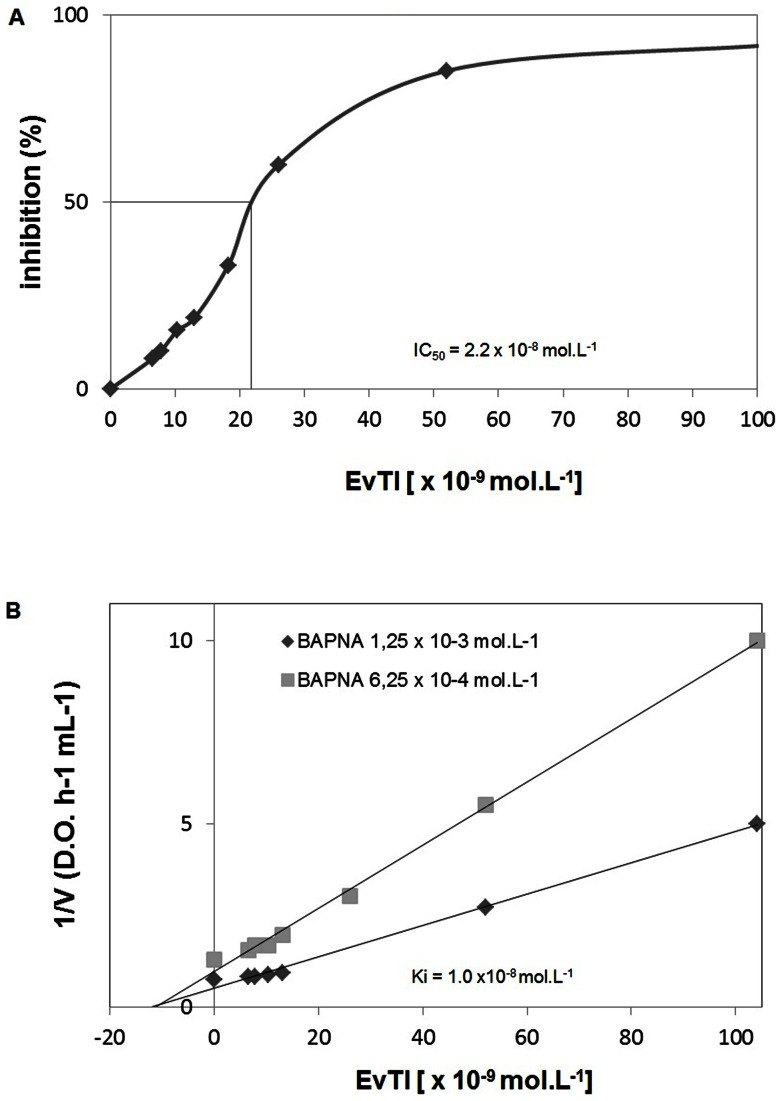
Determination of IC_50_ and Ki for a trypsin inhibitor purified from seeds of *Erythrina velutina* (EvTI). A. Inhibition curve of trypsin. IC_50_ was determined as EvTI concentration which inhibits 50% of trypsin activity. B. The inhibition constant (Ki) of EvTI on trypsin was determined by the method of Dixon (1979).

According to the titration curve shown in the Figure 2A, the EvTI IC_50_ for trypsin was about 2.2×10^−8^ mol.L^−1^. A sigmoidal profile of trypsin inhibition is observed. The plot analysis suggested that EvTI inhibits trypsin in a non-competitive manner with a K_i_ value of 1.0×10^−8^ mol.L^−1^ (Figure 2B).

### Analysis of the primary sequence and alignment

**Figure 3:**
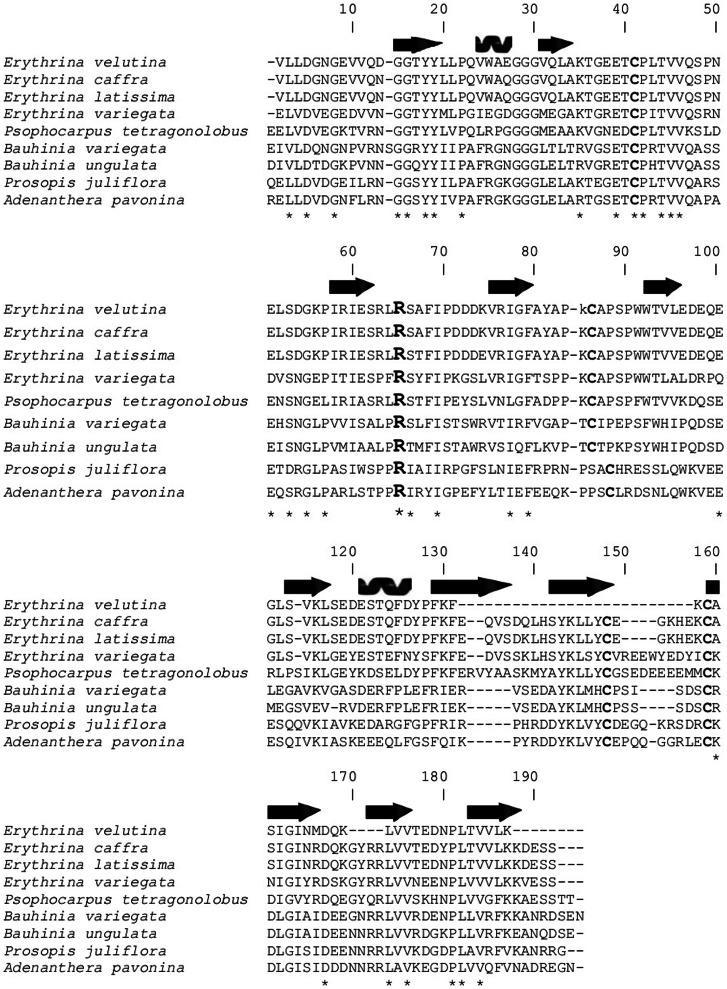
EvTI partial primary sequence alignment with sequences of known legume seeds Kunitz-type trypsin inhibitors: *E. velutina* (EvTI)*; E. caffra* (TSI) (gi 157833954)*; E. variegata* (ETIb) (gi 262753)*; Psophocarpus tetragonolobus* (WBTI) (gi 86450987)*; Bauhinia variegata* (BvcTI) (gi 15082208)*; Bauhinia ungulate* (gi 32363179)*; Prosopis juliflora* (PjTKI) (gi 243387)*; Adenanthera pavonina* (ApTKI) (gi 225058). Sequences deposited in the database of NCBI (National Center for Biotechnology Information) were compared using the BLASTp. The alignment was performed using Bio Edit software v 7.0.9.0 through the Clustal W multiple alignment tool.

Partial primary sequence of EvTI obtained by MALDI mass spectrometry was aligned with other Kunitz trypsin inhibitors from Papillionoideae subfamily, such as *Erythrina caffra* (gi 157833954), *E. variegate* (gi 262753), and *E. latissina* (gi 60392436), whose structures present high identity percentage of 82%, 81% and 51%, respectively. EvTI also showed similarity to other trypsin inhibitors from *Psophocarpus tetragonolobus* (gi 86450987), *Bauhinia variegate* (gi 15082208), *Bauhinia ungulate* (gi 32363179), *Prosopis juliflora* (gi 243387) *and Adenanthera pavonina* (gi 225058), but with low identity percentage ranging from 34 to 45% (Figure 3). This sequence corresponds to the protein fraction P2 obtained by reversed-phase chromatography. The peptide fragments sequenced for P1 were identical to those obtained for P2 fraction, but they did not cover a significant range of the sequence (data not shown). The distinctions between isoleucine and leucine are not possible by the mass spectrometry analysis and the choice is to make easier the alignment but has no experimental support. The percentage of amino acid sequence retrieved from the analysis by mass spectrometry MALDI-TOF was 84%.

### Cytotoxic and hemolytic EvTI activities

The cells of human peripheral blood did not suffer any cytotoxic effect when analyzed by blood cells counting or by hemolytic assay (results shown in Figure S2A and 2B).

### *In vitro* antibacterial activity of EvTI

EvTI was ineffective for *in vitro* inhibiting *E. coli* and *S. aureus* growth at all tested concentrations (2.0×10^−7^ to 2.7×10^−5^ mol.L^−1^ ), when compared with chloramphenicol (Figure S3).

### Anticoagulant activity

**Figure 4:**
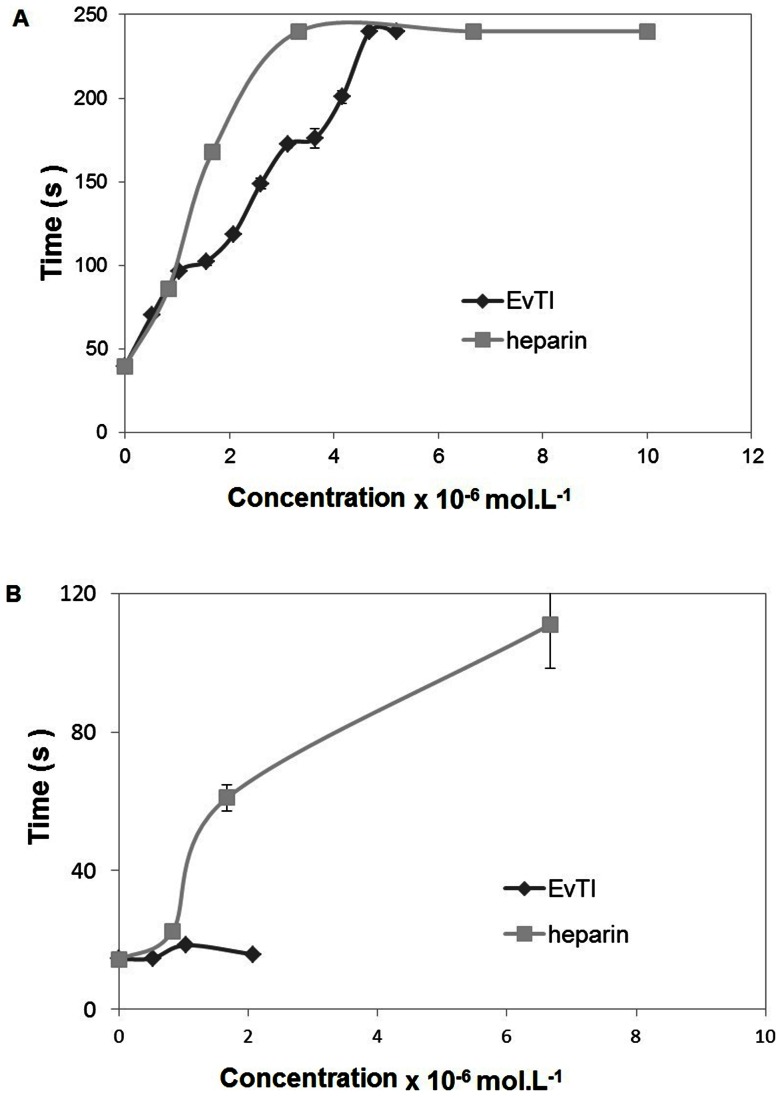
Effect of EvTI from *Erythrina velutina* seeds on the clot formation time using heparin as reference. A. Activated partial thromboplastin time (APTT). B. Prothrombin Time (PT).

According to the data obtained, EvTI was able to prolong the clotting time in a dose-dependent manner. At concentrations of 5.2×10^−7^ mol.L^−1^ the coagulation time was 71 s, being 1.8 times greater than the negative control group (40 s). The maximum action (240 s) was achieved with 4.7 μmol.L^−1^ EvTI and maintained constant at higher concentration of 5.2×10^−6^ mol.L^−1^ (Figure 4A). EvTI had no action on prothrombin time at neither of tested concentrations (Figure 4B).

### Evaluation of the migration of leukocytes into the peritoneal cavity

**Figure 5:**
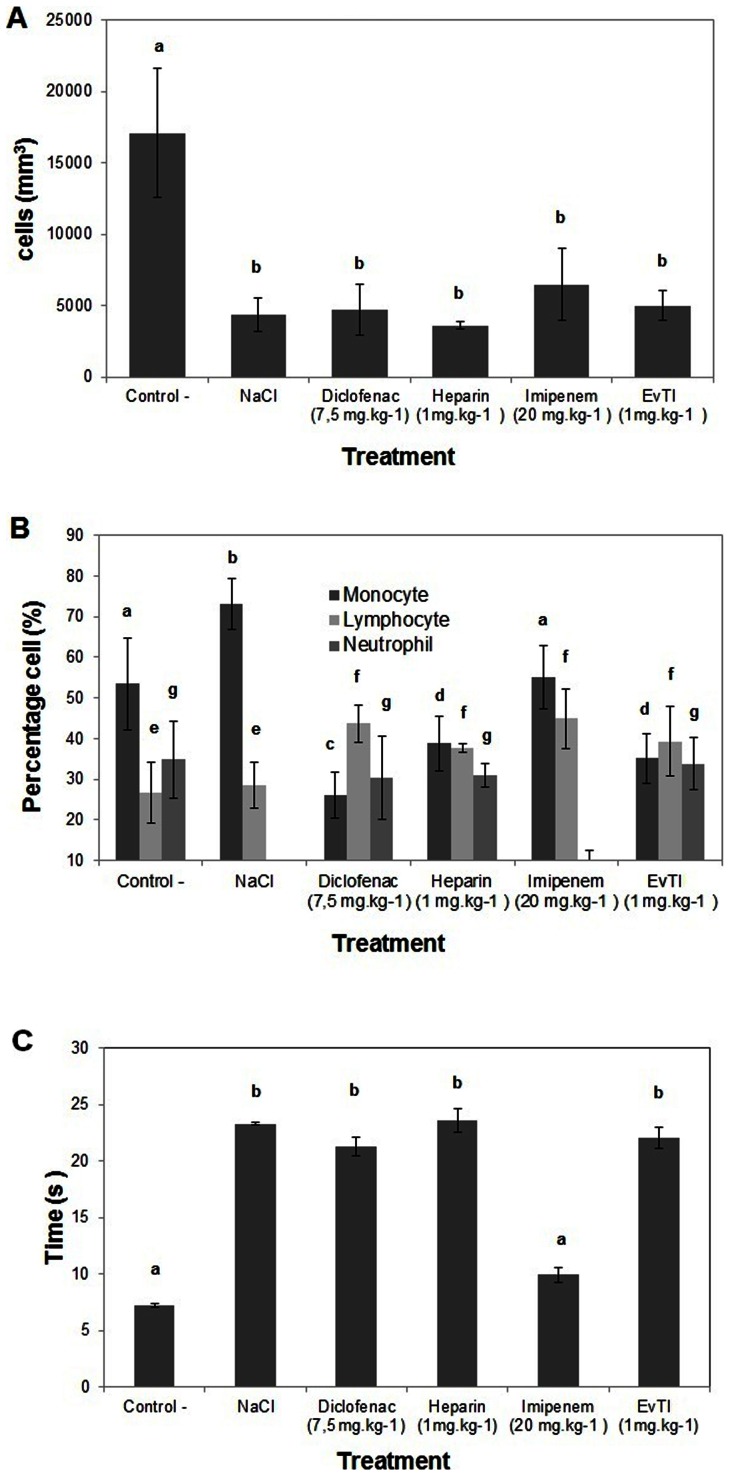
Anti-inflammatory and anticoagulant activities of EvTI purified from *Erythrina velutina* seeds. A. Effect of EvTI on leukocyte migration in septic mice. Groups: Control (0.9% (w:v) saline treatment and induction of sepsis), NaCl (0.9% (w:v) saline treatment, without induction of sepsis), Treatment with EvTI (1 mg.kg^−1^), diclofenac (7.5 mg.kg^−1^), heparin (1 mg.kg^−1^) and imipenem-1 (20 mg.kg^−1^) followed by induction of sepsis. B. Differential counts of peripheral blood leukocytes. C. Anticoagulant activity measured as activated partial thromboplastin time (APTT) in plasma of septic mice. Different letters indicate significantly different values according to ANOVA (p<0.05).

An intense cellular infiltration in the peritoneal cavity was observed six hours after sepsis induction, as can be seen in the group submitted to sepsis and treated with saline solution, which presented white-blood cell influx into the peritoneal cavity (17.000 cells.mm^−3^). This leukocyte influx into the peritoneal cavity is a characteristic symptom of inflammation (Figure 5A). For EvTI treatment at the dose of 1.0 mg.kg^−1^, one can observe a significant reduction in the influx of cells into the peritoneal cavity (a 71% decrease) compared with the group treated with saline, under sepsis condition. In groups treated with diclofenac, heparin or imipenem a significant reduction was observed in the influx of cells by 72%, 79% and 62%, respectively.

### Differential leukocyte count in peripheral blood

Blood cell populations that migrated into peripheral blood after 6 h treatment are shown in Figure 5B. It can be observed that the neutrophil percentage increased in whole blood of all sepsis induced groups, when compared with the control group, (except for the group treated with imipenem). In the groups treated with diclofenac, heparin, imipenem and EvTI, the monocyte migration decreased 64%, 47%, 25% and 52%, respectively. Moreover, in comparison with the control, lymphocytes were increased in the groups treated with diclofenac, heparin, imipenem and EvTI 1.6, 1.4, 1.7 and 1.4 times, respectively.

### Anticoagulant *ex vivo* activity in plasma of septic mice evaluated via the activated partial thromboplastin time (APTT) tests

The cecal ligation model and puncture (CLP) was also used to evaluate the effect of EvTI on the prolongation of clot formation in septic mice. It can be seen in Figure 5C that EvTI was able to restore the hemostatic levels when compared with control and no statistical difference in the groups treated with heparin and diclofenac. The group treated with imipenem had no effect on clotting.

### Evaluation of cytokine (TNF-α, IL-6, IFN-γ and IL-12) production and *in vivo* analyses

**Figure 6:**
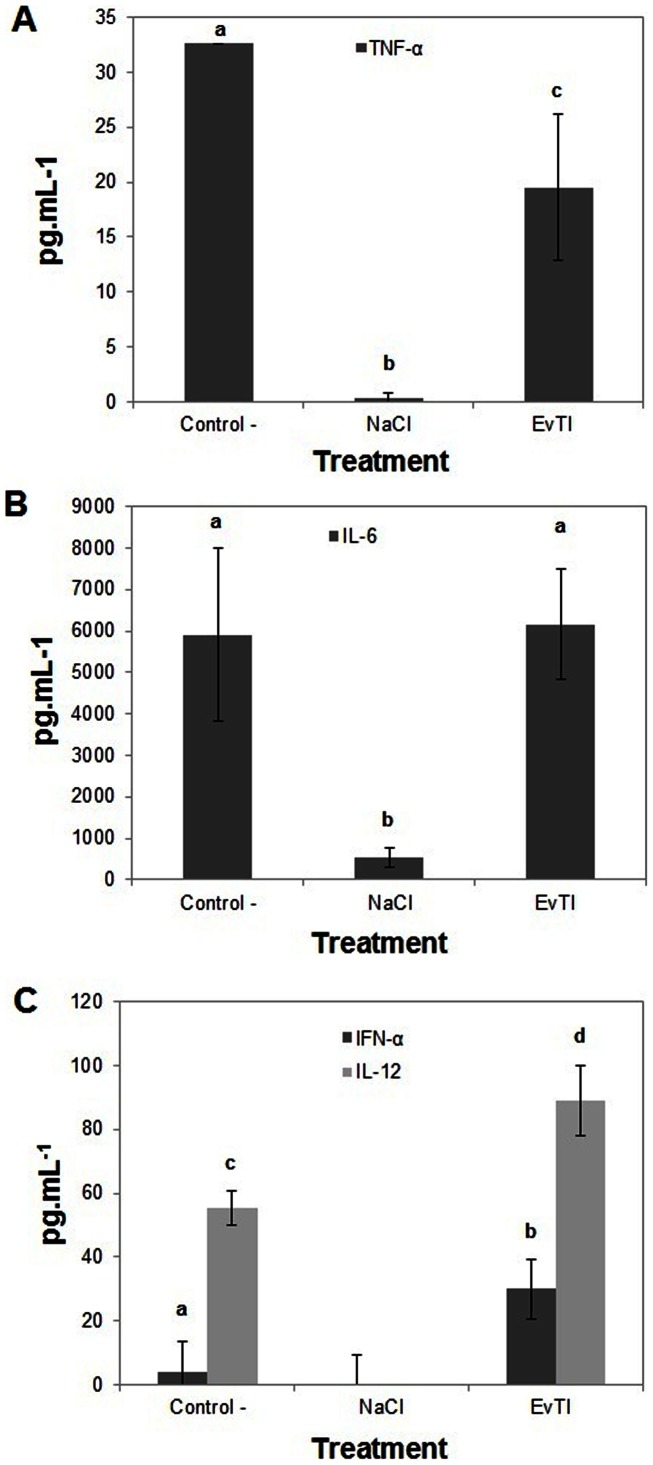
Determination of cytoKine levels in mice peritoneal fluid after 6 h of induction of sepsis in animals treated with EvTI purified from *Erythrina velutina* seeds. A, B, C. Dosage of TNF-α, IL-6, IFN-α and IL-12, respectively. Groups: Control – C (animals treated with 0.9%, w:v, NaCl and with induction of sepsis), NaCl (animals treated with 0.9%, w:v, NaClwithout induction of sepsis), EvTI (Treatment with 1 mg.kg^−1^ EvTI). The data represent the average of an experiment conducted with n = 8 in triplicate for each animal expressed as mean ± standard deviation. Asterisks indicate significantly different values according to ANOVA (p<0.05).

**Figure 7:**
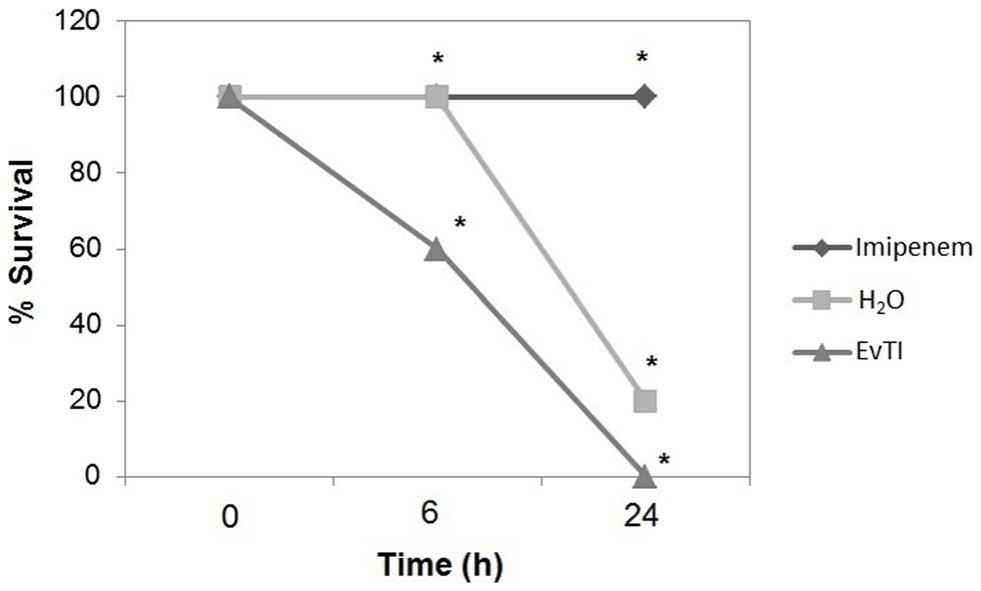
Influence of EvTI purified from *Erythrina velutina* seeds on survival of BALB/c mice subjected to polymicrobial sepsis. Mice were intraperitoneally inoculated with 25 μL of *Staphylococcus aureus* (5×10^9^ UFC.mL^−1^) and 25 μL *Escherichia coli* (5×10^7^ UFC.mL^−1^), and treated with EvTI at a dose of 10 mg.kg^−1^ for 24 h. Imipenem (20 mg.kg^−1^) was used as positive control and H_2_O was used as negative control.

The figure 6A shows that EvTI treatment in septic mice inhibited the TNF-α secretion by 41%. EvTI has no action on IL-6 release as shown in the Figure 6B. In the Figure 6C, IFN-α and IL-12 release in mice receiving EvTI were increased 7.6 and 1.6 fold, respectively, compared to control. IFN-α and IL-12 cytokines were not detected in the control group (without inflammation) and only small amounts of TNF-α and IL-6 were detected in this group. Additionally, the presence of nitric oxide was not detected in peritoneal fluid in all groups (data not shown). Finally it is important to cite that EvTI was unable to protect the mice at polymicrobial sepsis model, do not improving the rates survival (Figure 7).

## Discussion

The *in vitro* and *in vivo* effects on pathogens and the various therapeutic possibilities in the treatment of a wide range of human health disorders have made peptidase inhibitors important targets of research. Many studies led to the identification and purification of inhibitors from various animal and plant sources, as well as their biochemical, three-dimensional structures, specificities and mechanisms of inhibition characterizations [15], [54], [55].

The inhibitors purified from seeds of *E. velutina* showed molecular masses of 19,228.16 Da and 19,210.48 Da. The molecular weights obtained for the trypsin inhibitor purified from *E. velutina* are in agreement with those determined for the Kunitz family of inhibitors with molecular mass ranging from 18 to 26 kDa, such as ECTI (*Enterolobium contortisiliquum* seeds trypsin inhibitor), BvcTI (*Bauhinia variegata* trypsin inhibitor), DMTI (*Dimorphandra mollis* seeds trypsin inhibitor) AETI (*Archidendron ellipticum* seeds trypsin inhibitor), CBTI-2 (*Caesalpinia bonduc* seeds trypsin inhibitor) and ILTI (*Inga laurina* seeds trypsin inhibitor) [19], [56]–[60]. It is important to point out that the mass spectrometry analyses show the possible existence of other inhibitor isoforms since small tags of other sequences not presented here were found. Thus, one must be cautious that the protein characterization reported here is a first step to the full knowledge of peptidase inhibitors in *E. velutina*.

After purification, EvTI showed higher specificity for the trypsin and factor Xa when compared to other enzymes such as chymotrypsin and thrombin and a reasonable recovery yield, so this inhibitor was selected for further analysis of structure and function. The Kunitz-type classification of EvTI was initially suggested by molecular mass, and after confirmed by MS sequencing. This classification was corroborated by high identity of structures observed between EvTI and known Kunitz inhibitors from *E. cafra*, *E. latissima* and *E. variegata*, being the identity percentages of 82%, 81% and 51%, respectively. Furthermore, the high functional stability of EvTI is typical for the Kunitz-type inhibitor family [61]. EvTI showed no reduction in its inhibitory activity when exposed to temperatures up to 100°C for 30 min and pH variations from 2 to 12 range. Similar data were obtained for other known inhibitors, such as the inhibitor present in *Poecilanthe parviflora* seeds (PPTI) and the chymotrypsin inhibitor from *Erythrina velutina* seeds (EvCI), which maintained their activities after exposure to temperature of 100°C for 30 min [62]. Inhibitors present in *Crotalaria pallida* (CpaTI) and that of *Pithecellobium dumosum* (PDKI) seeds maintained their activities in a pH range of 2–12 [63], [64]. In agreement with those authors, the high functional stability of EvTI to pH and temperature variation can be explained by the presence of intramolecular disulfide bonds in its structure, that is typical in Kunitz-type inhibitors structures.

EvTI is an non-competitive trypsin inhibitor similar to other trypsin Kunitz-like inhibitors, such as AETI from *Archidendron ellipticum* seed [59], APTI from *Adenanthera pavonina* seed [65] and TTI from tamarind seed [66]. Otherwise, the inhibition mechanism of EvTI is different of a Kunitz-type inhibitor PdKI2 from *Pithecelobium dumosum* seed, which inhibits trypsin by competitive mode [63]. The IC_50_ of EvTI for trypsin was 2.2×10^−8^ mol.L^−1^ (0.42 µg.mL^−1^), that was less potent than urinary trypsin inhibitor (IC_50_ of 6.44×10^−10^ mol.L^−1^) [67], and lower than trypsin inhibitor from *Sapindus saponaria* seeds L (SSTI2 IC_50_ 8.3×10^−8^ mol.L^−1^), and than purified trypsin inhibitors from *Phaseolus vulgaris* (IC_50_ = 6.0×10^−7^ mol.L^−1^) and from *Glycine max* (IC_50_ = 1.9×10^−5^ mol.L^−1^) [68], [69]. These results indicate that EvTI is effective on inhibiting trypsin like other inhibitors described in the literature. The inhibition constant (K_i_) for EvTI was 1.0×10^−8^ mol.L^−1^. Comparing the Ki values with other inhibitors of the Kunitz family, it was observed that the EvTI has similar affinity for trypsin than one inhibitor found in *E. variegata* IATT IATT-N12A and higher affinity than a second inhibitor of the same species, which present K_i_ values of 2.0×10^−8^ and 5.2×10^−6^ mol.L^−1^, respectively. Similar Ki values were also found in the ILTI from *Inga laurina* seeds [57] and DMTI from *Dimorphandra mollis* seeds [58] with K_i_ of 6.0×10^−9^ and 5.3×10^−9^ mol.L^−1^, respectively. Lower K_i_ values were found in AETI [59] and CBTI-2 [60] with Ki of 2.4×10^−10^ and 2.7×10^−10^ mol.L^−1^, respectively. The value of K_i_ determined for EvTI is thus within the characteristic range described for the Kunitz family of inhibitors. The Kinetic parameters Ki and IC_50_ observed for EvTI together with molecular weight and sequencing data corroborate their definition as a Kunitz-type inhibitor.

After the biochemical characterization of EvTI, its pharmacological properties were explored. The use of peptidase inhibitors is already part of clinical practice for the treatment of certain disorders in the coagulation cascade [28], [29]. The Kunitz-type inhibitors are well characterized compounds that may block serine peptidase involved in platelet aggregation, blood coagulation, fibrinolysis and inflammation [70]. Before evaluating the pharmacological properties, EvTI cytotoxicity was assessed in human peripheral blood cells. EvTI showed neither hemolytic nor toxic effects on blood cell populations even at high inhibitor doses. Similar results were found for trypsin inhibitors of *Coccinia grandis* [71] and xb-KTI [5] from *Xanthosoma blandum*, suggesting a valuable and desirable low toxicity of EvTI.

EvTI was able to increase the clotting time to 240 s when measured by APTT assays. Meanwhile, this inhibitor showed no action on prothrombin time (PT). These results show that EvTI seems to act specifically on the intrinsic pathway of coagulation. EvTI inhibited factor Xa by 80%, but showed no inhibitory activity toward thrombin. Similar results were found for ECTI (trypsin inhibitor from *Enterolobium contortisiliquum* seeds) [19], which also inhibited clot formation, but did not inhibit Xa. EvCI (chymotrypsin inhibitor from seeds of *E. velutina*) [18] (6 µg) also inhibited the clot formation by about 80 s and factor Xa. Trypsin inhibitor from *Leucaena leucocephala* (LITI) by the highest assayed dose tested (12 µg) also inhibited clot formation at 60 s [72]. However, EvTI inhibited clot formation by 240 s with only 18 µg. The results of EvTI inhibition activity on clot formation are commonly described in the literature for other known inhibitors and are similar to that observed for commercial heparin. These results indicate that EvTI may be a promising tool in the development of new anticoagulant compounds.

There are many studies evidencing a close relationship between inflammation and coagulation [23], [25]–[27], [73]. Virtually all situations that lead to a systemic inflammatory response are associated with some degree of coagulation activation. It is known that inflammatory cytokines such as TNF-α and IL-6 are potent inflammatory mediators that activate coagulation factors [73]. In the early stages of sepsis, the coagulation system and platelets are activated and may cause thrombocytopenia and coagulopathy [74]. Systemic coagulation activation not only promotes fibrin deposition and thrombosis, which is considered pathologically more important and is strongly linked to the development of multiple organ dysfunction (MOD), but also occurs in sepsis consumption and consequent depletion of clotting factors and platelets, which often results in hemorrhagic manifestations [74], [75].

Although there are many controversies in sepsis investigations, it is agreed that the process and treatment of this disease are extremely complex. Therefore, many studies using animal models have been carried out in order to understand the pathophysiological responses of sepsis, thus simulating the observed changes in human sepsis [76]. Several models have been used, but the model of sepsis induced by ligation and perforation of the cecum (CLP) has been widely used to investigate various aspects of sepsis and septic shock, because this model resembles the characteristics and progression of human sepsis [77], [78]. Since this model is also an important tool for evaluation of a potential drug for the treatment of sepsis, it was used in this study.

Animals subjected to this experimental model showed characteristic sepsis symptoms within one hour after surgery, including piloerection, lethargy, exudate over the eyes and nose, abdominal enlargement and leukocytosis (over 12000.mm^−3^). These symptoms were similar to those of several studies reported in the literature [77], [78]. In this experimental model of sepsis, EvTI showed anti-inflammatory characteristics by reducing the neutrophil infiltration by 71%, 6 h after induction of inflammation. Similar results were found for the inhibitor of chymotrypsin (EvCI), which reduced the number of leukocytes recruited to the site of inflammation by 87% [18]. Regarding release of inflammatory cytokines, EvTI was able to decrease the levels of TNF-α by 41% (inflammatory cytokine). This is an important result, since some patients may early evolve to death due to the intense inflammatory reaction triggered by excessive inflammatory cytokine secretion [79]. The suppression of the TNF-α release is another important factor, since it is considered a potent mediator in sepsis from various evidences, since it is first cytokine that appears in the circulation in human sepsis. Studies have shown that the administration of this cytokine induces a syndrome in animals with characteristic of sepsis, and finally treatment with anti-TNF antibody protects against the lethal effects of endotoxins in animal models [80]. IL-6 and IL-1 leads to activation of the coagulation system though the hydrolysis of factors IX and X. Furthermore, IFN-α and IL-12 release increased by 7.6 and 1.6 times, respectively, when compared to control. A study by [81] shows that IL-12 seems to be a potent immune stimulant and inducer of Th1 response and that there was a reduction in mortality when Il-12 is administered in mice with sepsis induced by CLP.

One of the practices adopted for the treatment of sepsis or septic shock is the use of antibiotics and drugs that interfere with cardiovascular changes, but do not act effectively on the inflammatory response, and this may be one reason for the high mortality of patients with septic shock [80]. Thus, EvTI in the treatment of sepsis should be employed in combination with antibiotics already used in clinical practice, because EvTI acts longer on cellular mechanisms than on microorganisms. Nevertheless, EvTI showed atypical behavior, since a low antibacterial activity was observed. Moreover, this inhibitor was unable to induce significant IL-12 secretion, as this cytokine is secreted at low basal levels. EvTI stimulated IFN-α release and acted specifically in reducing the levels of TNF-α, leukocyte migration and inhibiting coagulation factor Xa. The administration of peptidase inhibitors for enzymes involved in the coagulation cascade could inhibit coagulation, diminishing the mortality caused by a diffuse activation. This activation may lead to vessel occlusion, which is a consequence of fibrin deposition in sepsis, and may also lead to the induction or modulation of production of cytokines or inflammatory modulators.

There was no protective effect of EvTI on survival of mice subjected to polymicrobial sepsis. The action of EvTI in sepsis model induced by ligation and perforation of the cecum can be attributed to the reduction in leukocyte migration, reduced levels of potent inflammatory mediators and anticoagulant action of EvTI, acting specifically on Factor Xa and preventing disseminated intravascular coagulation in an experimental model of sepsis. Nevertheless, these effects are not enough for an *in vivo* efficacy of the inhibitor but a combination with other drugs like antibiotics could be assayed.

The results for the EvTI can contribute to a better understanding of the pathophysiology of sepsis and its treatment. Moreover, the bioprospection of unusual and potent peptidase inhibitors for treatment of different diseases is extremely important for drug development. Therefore, purification and structural characterization of a functional trypsin inhibitor from *Erythrina velutina* (EvTI) seeds with capability to interfere in different biological processes such as blood coagulation and inflammation, without presenting cytotoxicity, are important data that may lead to new compounds being obtained, which can have potential for drug development in the treatment of various coagulation disorders.

## Conclusions

The trypsin inhibitor from seeds of *Erythrina velutina* (EvTI) was purified and characterized, and its multifunctional activities were evaluated. EvTI showed anticoagulant activity and modulating activity in sepsis induced by cecal ligation and puncture. Furthermore, relevant cytotoxic and antimicrobial activities were not observed. These results contribute to a better understanding of the use of peptidase inhibitors in inflammation and coagulation in an experimental model of sepsis, contributing to a better understanding of the pathophysiology of sepsis given that both processes are a common cause of death in humans. Since sepsis is a syndrome that requires emergency intervention due to increasing incidence and high mortality, the data reported here shed some light on the place of Kunitz-type inhibitors in sepsis. The complex pathophysiology of sepsis, involving immunity, inflammatory mechanisms and the coagulation cascade, culminating in a state of intense alteration of homeostasis in its more advanced stages, draws attention to the urgent need for more studies involving therapeutic agents that modulate such disorders.

## Supporting Information

Figure S1 **Stability of EvTI purified from** ***Erythrina velutina*** **seeds as a function of pH and temperature.** A. Stability at pH variation. B. Thermal stability. EvTI (2.6×10^−7^ mol.L^−1^) was pre-incubated for 30 min at different temperatures or pH at 37°C. The inhibitory activity on trypsin (13×10^−6^ mol.L^−1^) was determined by using BApNA (1,25.10^−3^ mol.L^−1^) as substrate.
(TIF)

Figure S2 **Cytotoxicity of trypsin inhibitor purified from seeds of** ***Erythrina velutina*** **(EvTI) and flow cytometry of human peripheral blood in the presence and absence of EvTI.** A. Human peripheral blood in the absence of EvTI, andin the presence of EvTI (2.6×10^−8^ to 1.3×10^−6^ mol.L^−1^). Analysis of cytotoxic effects on peripheral blood EvTI total. Absence of inhibitor control. LYM (%), percentage of lymphocytes; WBC (×10^3^/μL) RBC (×10^6^/μL) and PKT (×10^3^/μL), white blood cell count, platelets and erythrocytes, respectively. B) Evaluation of EvTI hemolytic effect on human erythrocytes. Increasing concentrations of the inhibitor were used (3 to 50 μg.μL^−1^ or 1.6×10^−8^ mol.L^−1^ to 2.6×10^−9^ mol.L^−1^). As positive and negative control phosphate buffered saline (PBS), pH 7.2, and Triton X-100 1% (by volume) were used, respectively. Different letters indicate significantly different values according to ANOVA (p<0.05).
(TIF)

Figure S3 **Evaluation of antibacterial activity of trypsin inhibitor purified from seeds of** ***Erythrina velutina*** **(EvTI).** A) Effect of different concentrations of EvTI on the growth of *Escherichia coli*. B) Effect of different concentrations on the growth of *Staphylococcus aureus*. EvTI of 2.0×10^−7^ mol.L^−1^ to 5.3×10^−6^ mol.L^−1^. The bacterial culture in PBS represents the maximum growth. Chloramphenicol was used as positive control. Assays were performed in triplicate and data are representative of the average of the results obtained (mean ± SD).
(TIF)
