# Supplementary figures and images for: Characterization and Pharmacological Properties of a Novel Multifunctional Kunitz Inhibitor from Erythrina velutina Seeds
Source: PLoS One. 2013 May 28;8(5):e63571. doi: 10.1371/journal.pone.0063571 (PMC3666885; doi:10.1371/journal.pone.0063571)

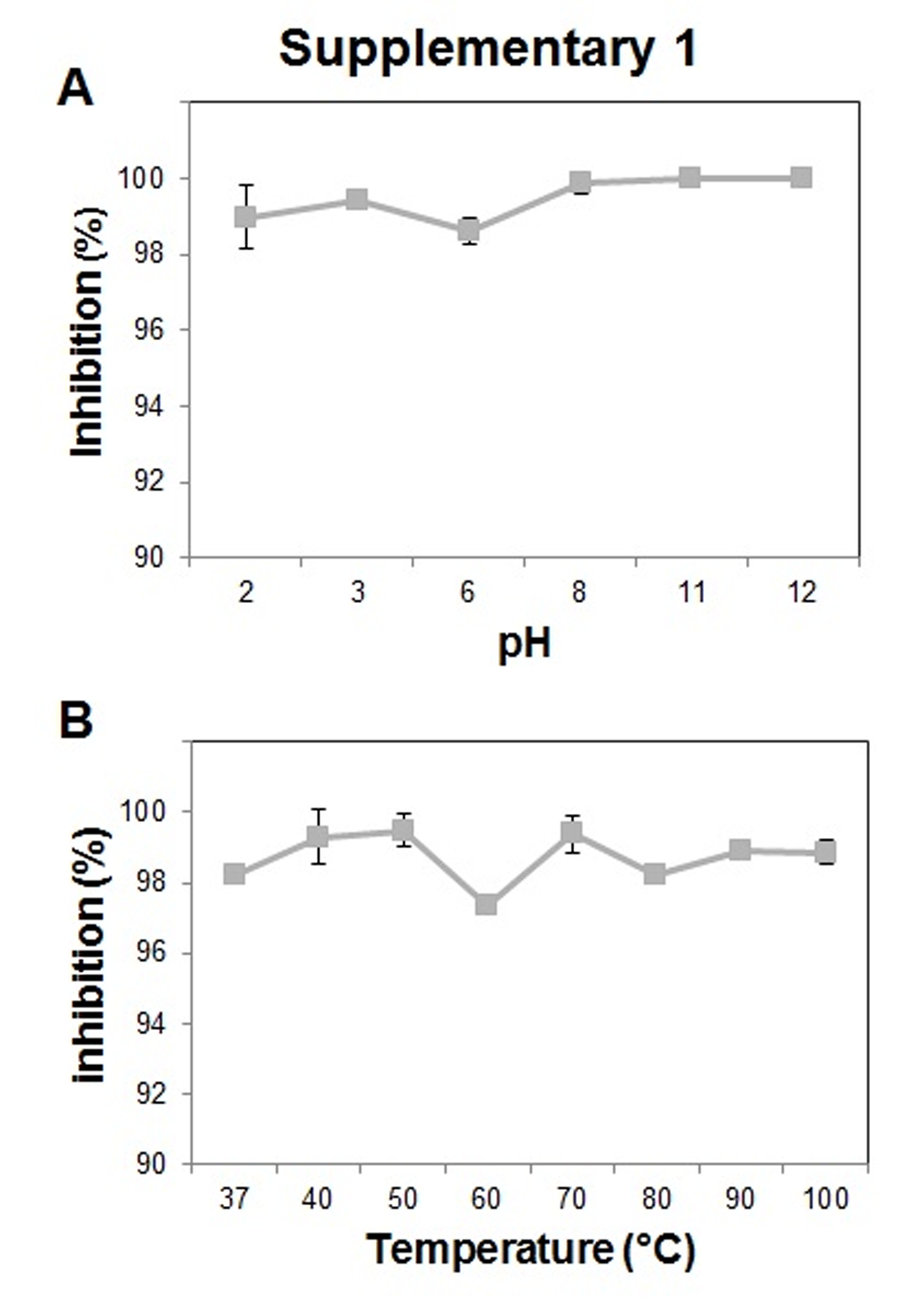

Supplement: Figure S1 — Stability of EvTI purified from Erythrina velutina seeds as a function of pH and temperature. A. Stability at pH variation. B. Thermal stability. EvTI (2.6×10^−7^ mol.L−1) was pre-incubated for 30 min at different temperatures or pH at 37°C. The inhibitory activity on trypsin (13×10−6 mol.L−1) was determined by using BApNA (1,25.10−3 mol.L−1) as substrate. (TIF) [file pone.0063571.s001.tif]

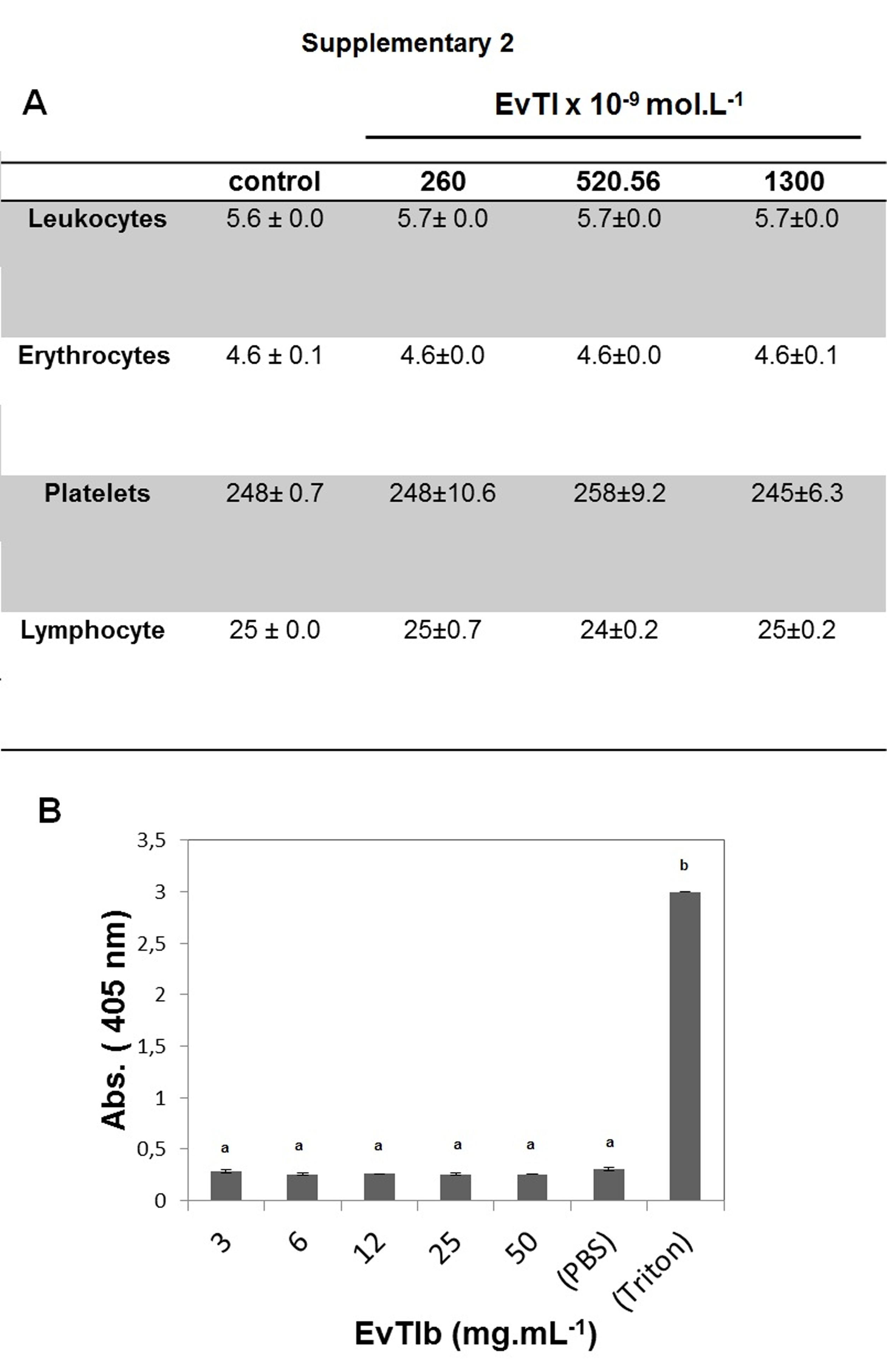

Supplement: Figure S2 — Cytotoxicity of trypsin inhibitor purified from seeds of Erythrina velutina (EvTI) and flow cytometry of human peripheral blood in the presence and absence of EvTI. A. Human peripheral blood in the absence of EvTI, andin the presence of EvTI (2.6×10^−8^ to 1.3×10^−6^ mol.L−1). Analysis of cytotoxic effects on peripheral blood EvTI total. Absence of inhibitor control. LYM (%), percentage of lymphocytes; WBC (×103/μL) RBC (×106/μL) and PKT (×103/μL), white blood cell count, platelets and erythrocytes, respectively. B) Evaluation of EvTI hemolytic effect on human erythrocytes. Increasing concentrations of the inhibitor were used (3 to 50 μg.μL−1 or 1.6×10^−8^ mol.L−1 to 2.6×10^−9^ mol.L−1). As positive and negative control phosphate buffered saline (PBS), pH 7.2, and Triton X-100 1% (by volume) were used, respectively. Different letters indicate significantly different values according to ANOVA (p<0.05). (TIF) [file pone.0063571.s002.tif]

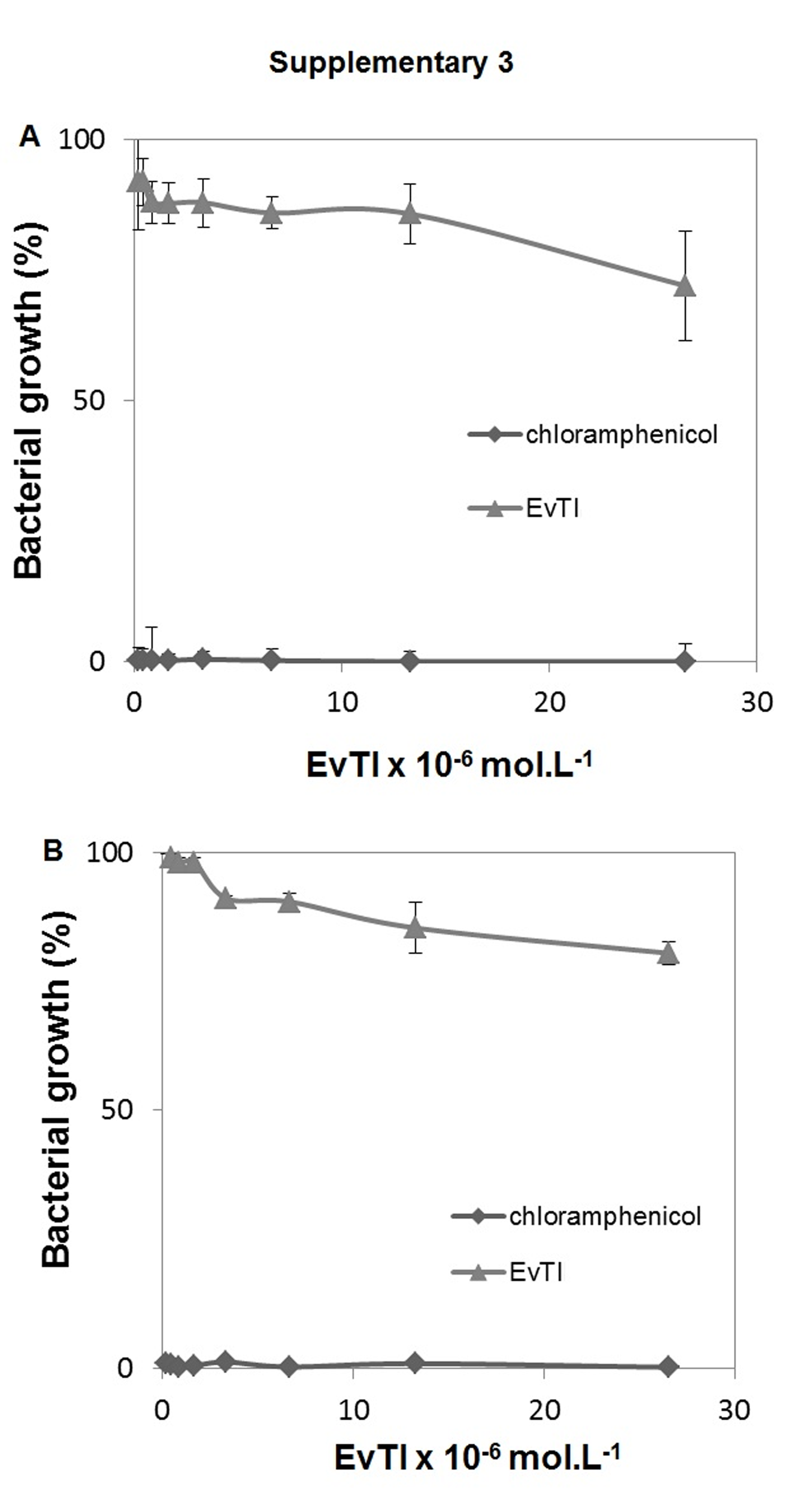

Supplement: Figure S3 — Evaluation of antibacterial activity of trypsin inhibitor purified from seeds of Erythrina velutina (EvTI). A) Effect of different concentrations of EvTI on the growth of Escherichia coli. B) Effect of different concentrations on the growth of Staphylococcus aureus. EvTI of 2.0×10^−7^ mol.L−1 to 5.3×10^−6^ mol.L−1. The bacterial culture in PBS represents the maximum growth. Chloramphenicol was used as positive control. Assays were performed in triplicate and data are representative of the average of the results obtained (mean ± SD). (TIF) [file pone.0063571.s003.tif]
